# Cerium(III) dihydroxidohexa­oxidotetra­borate chloride

**DOI:** 10.1107/S1600536812002875

**Published:** 2012-01-31

**Authors:** Wei Sun, Biao-Chun Zhao, Ya-Xi Huang, Jin-Xiao Mi

**Affiliations:** a Department of Materials Science and Engineering, College of Materials, Xiamen University, Xiamen 361005, Fujian Province, People’s Republic of China

## Abstract

The crystal structure of the title compound, Ce[B_4_O_6_(OH)_2_]Cl, is built from polyborate sheets parallel to the (001) plane. These sheets stack along the [001] direction and are linked by Ce atoms exhibiting an CeO_8_Cl_2_ coordination sphere. O—H⋯O and O—H⋯Cl hydrogen bonds additionally stabilize the structural set-up. The polyborate sheet is made up of zigzag borate chains running along the [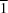
10] direction. These zigzag chains are inter­connected by shared O-vertices, resulting in a two-dimensional layer with nine-membered rings. All B and O atoms (except for the terminal OH atoms) lie in the nearly planar sheets of polyborates, leading to their isotropic atomic displacement parameters being significantly smaller than usual. This may be attributed to the fact that the atomic displacement parameters correlate not only with their atomic masses but with their coordination environments also.

## Related literature

For background to borate compounds and their applications, see: Burns *et al.* (1995 ▶); Chen *et al.* (1985 ▶); Zhao *et al.* (1990 ▶); Sun, Sun *et al.* (2010 ▶); Sun, Zhou *et al.* (2010 ▶). For isotypic structures, see: Belokoneva *et al.* (2002 ▶) for *Ln*[B_4_O_6_(OH)_2_]Cl (*Ln* = Pr, Nd).

## Experimental

### 

#### Crystal data

Ce[B_4_O_6_(OH)_2_]Cl
*M* *_r_* = 348.83
Monoclinic, 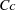

*a* = 6.5169 (11) Å
*b* = 11.245 (2) Å
*c* = 9.7575 (17) Å
β = 105.284 (3)°
*V* = 689.8 (2) Å^3^
*Z* = 4
Mo *K*α radiation
μ = 7.00 mm^−1^
*T* = 295 K
0.16 × 0.07 × 0.03 mm

#### Data collection

Bruker SMART APEX CCD area-detector diffractometer
Absorption correction: multi-scan (*SADABS*; Bruker, 2001 ▶) *T*_min_ = 0.401, *T*_max_ = 0.818
2825 measured reflections
1532 independent reflections
1523 reflections with *I* > 2σ(*I*)
*R*_int_ = 0.022

#### Refinement

*R*[*F* ^2^ > 2σ(*F* ^2^)] = 0.020
*wR*(*F* ^2^) = 0.051
*S* = 1.00
1532 reflections
134 parameters
10 restraints
All H-atom parameters refined
Δρ_max_ = 0.53 e Å^−3^
Δρ_min_ = −1.64 e Å^−3^
Absolute structure: Flack (1983 ▶), 716 Friedel pairs
Flack parameter: 0.034 (19)

### 

Data collection: *SMART* (Bruker, 2001 ▶); cell refinement: *SAINT* (Bruker, 2001 ▶); data reduction: *SAINT*; program(s) used to solve structure: *SHELXS97* (Sheldrick, 2008 ▶); program(s) used to refine structure: *SHELXL97* (Sheldrick, 2008 ▶); molecular graphics: *DIAMOND* (Brandenburg, 2011 ▶) and *ATOMS* (Dowty, 2004 ▶); software used to prepare material for publication: *SHELXL97*.

## Supplementary Material

Crystal structure: contains datablock(s) I, global. DOI: 10.1107/S1600536812002875/fj2495sup1.cif

Structure factors: contains datablock(s) I. DOI: 10.1107/S1600536812002875/fj2495Isup2.hkl

Additional supplementary materials:  crystallographic information; 3D view; checkCIF report

## Figures and Tables

**Table 1:** Hydrogen-bond geometry (Å, °)

*D*—H⋯*A*	*D*—H	H⋯*A*	*D*⋯*A*	*D*—H⋯*A*
O8—H1⋯O1^i^	0.82 (2)	2.43 (7)	2.859 (5)	114 (6)
O8—H1⋯Cl1^ii^	0.82 (2)	2.52 (5)	3.219 (4)	145 (7)
O7—H2⋯O4^ii^	0.83 (2)	2.13 (6)	2.860 (6)	148 (10)
O7—H2⋯Cl1^ii^	0.83 (2)	2.81 (11)	3.220 (4)	112 (9)

Symmetry codes: (i) 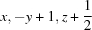; (ii) 
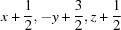
.

**Fig. 1.**
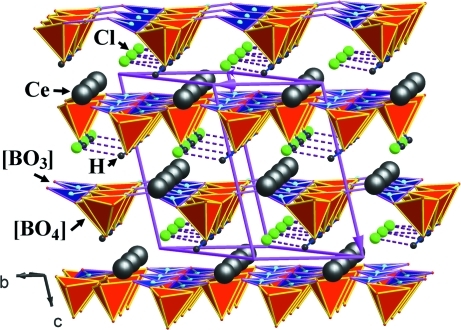
Crystal structure of Ce(B4O6(OH)2)Cl. ([BO4] drawn in red tetrahedra; [BO3] in blue triangular groups; Ce in black spheres; Cl in green spheres; H in small black balls).

**Fig. 2.**
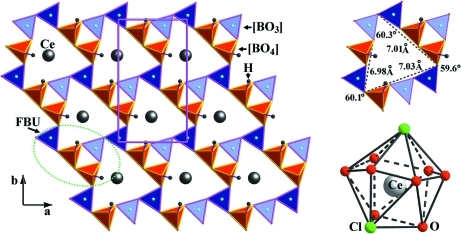
Left: a two-dimensional sheet with 9-membered rings, the fundamental building block (FBB) marked by a dash-line ellipse; Right upper: a 9-membered ring with a nearly equilateral triangular motif; Right down: coordination environment of Ce in a 1-6-3 crown-shaped polyhedron.

**Fig. 3.**
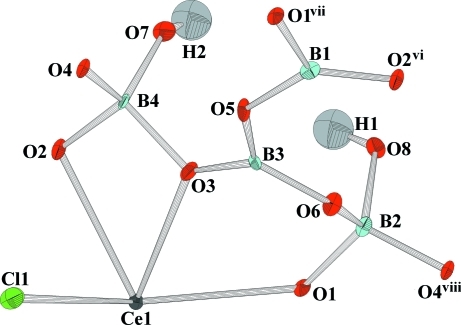
Coordination environment of metal atoms, with displacement ellipsoids drawn at the 50% probability level (symmetry codes: (vi) x–1/2, y–1/2, z; (vii) x–1, y, z; (viii) x + 1/2, y–1/2, z)

## supplementary crystallographic information

### Comment

Borate compounds have been extensively studied due to their variety of fundamental building blocks and crystal structure types (Burns *et al.*, 1995), as well as their successful industry applications as nonlinear optical materials, *e.g.*β-Ba(B_2_O_4_) (BBO) (Chen *et al.*, 1985) and LiB_3_O_5_ (LBO) (Zhao *et al.*, 1990). Besides alkali metals and alkaline-earth metals (Sun, Sun *et al.*, 2010), rare-earth elements become highlighted with being introduced into the borate system in order to obtain multifunctional materials with the distinctive luminescence properties of rare-earth elements. Belokoneva *et al.* (2002) claimed that *Ln*(B_4_O_6_(OH)_2_)Cl (*Ln* = Pr, Nd) exhibits excellent nonlinear optical properties. But the atomic coordinates of its cerium analogue have not been reported until now. Herein, we report the crystal structure of Ce(B_4_O_6_(OH)_2_)Cl determined from single-crystal X-ray diffraction data, including the sites of hydrogen atoms.

The crystal structure of Ce(B_4_O_6_(OH)_2_)Cl is characterized as a layered strcuture of polyborate sheets parallel to (001)(Fig. 1 & 2). In the structure boron atoms have two types of polyhedral coordinations. One is 3-coordinated by oxygen atoms, forming a triangular planar [BO_3_] group. The other is 4-coordinated to three O-atoms and one hydroxyl group to form a [BO_3_(OH)] tetrahedron (Fig. 3). Both [BO_3_] group and [BO_3_(OH)] polyhedron link to three neighbouring borate groups *via* their common O-corners except for OH terminals. Two [BO_3_(OH)] tetrahedra and two triangular planar [BO_3_] groups compose a borate tetramer as a fundamental building block (FBB) (Fig. 2) (Burns *et al.*, 1995). The FBBs share their common oxygen vertices to form a zigzag borate chain running along the [110] direction. The zigzag chains are further interconnected with each other by sharing their common O-corners, resulting in a two-dimensional layer with 9-membered rings within the layer. The 9-membered ring has a nearly equilateral (about 7.00 Å) triangular motif (Fig. 2). The Ce atoms just reside at the center of 9-membered rings and adopt a 10-coordination with the surrounding eight oxygen and two chlorine atoms, forming a 1-6-3 crown-shaped polyhedron (Fig. 2). The two-dimensional layers stack along the [001] direction and are linked by Ce and Cl atoms as well as hydrogen bonds to form the three-dimensional crystal structure (Fig. 1). It is interesting that all boron and oxygen atoms (except for OH terminals) lying in the nearly planar sheets of polyborates lead to their isotropic atomic parameters significantly smaller than as-expected usually. For example, the isotropic atomic parameters of boron atoms are distributed in the range of 0.0062 (10) to 0.0085 (10) Å^2^, significantly smaller than that of chlorine atom (0.0130 (2) Å^2^). This may give a hint that the atomic displacement parameters correlate not only with their atomic masses but with their coordination environments also. However the standard uncertainties of atomic displacement parameters do have close correlation with their atomic masses of individual elements. As-observed in the title compound, standard uncertainties of atomic displacement parameters of all atoms distribute as-expected in the sequence of Ce, Cl, O, B and H, increasing while their atomic masses decrease.

### Experimental

The title compound, Ce(B_4_O_6_(OH)_2_)Cl was synthesized by using a hydrothermal method during our systematically exploiting rare earth borates (Sun, Zhou *et al.*, 2010). Typically, a mixture of Ce_2_O_3_ (0.33 g), CrCl_3_.6H_2_O (0.80 g), H_3_BO_3_ (2.00 g) and 2 ml distilled water with molar ratio of Ce: Cl: B = 1: 9: 32 was prepared, and transferred into a Teflon-lined stainless-steel autoclave (30 ml in volume), then heated to and kept at 468 K for three days. Transparent, colorless crystals of the title compound were obtained by filtration, rinsed with distilled water several times, and dried in desiccators. Optical examination and powder X-ray diffraction (PXRD) analyses were used to identify the phases of the solid products.

### Refinement

The hydrogen positions were obtained from the difference Fourier map and refined *via* fixing the bond distance of *d*(OH) = 0.82 (2)Å with the donator of coordinated oxygen atoms. Moreover a common variable was set for the isotropic atomic displacement parameters of all hydrogen atoms during the refinement.

### Crystal data

**Table:**

Ce[B_4_O_6_(OH)_2_]Cl	*F*(000) = 644
*M**_r_* = 348.83	*D*_x_ = 3.359 Mg m^−^^3^
Monoclinic, *C**c*	Mo *K*α radiation, λ = 0.71073 Å
Hall symbol: C -2yc	Cell parameters from 2825 reflections
*a* = 6.5169 (11) Å	θ = 3.6–28.0°
*b* = 11.245 (2) Å	µ = 7.00 mm^−^^1^
*c* = 9.7575 (17) Å	*T* = 295 K
β = 105.284 (3)°	Prism, colorless
*V* = 689.8 (2) Å^3^	0.16 × 0.07 × 0.03 mm
*Z* = 4	

### Data collection

**Table:**

Bruker SMART APEX CCD area-detector diffractometer	1532 independent reflections
Radiation source: fine-focus sealed tube	1523 reflections with *I* > 2σ(*I*)
graphite	*R*_int_ = 0.022
1800 images,φ = 0, 90, 180°, χ = 54.74°, Δω=0.3°, Exp time: 15 s. scans	θ_max_ = 28.0°, θ_min_ = 3.6°
Absorption correction: multi-scan (*SADABS*; Bruker, 2001)	*h* = −8→8
*T*_min_ = 0.401, *T*_max_ = 0.818	*k* = −14→14
2825 measured reflections	*l* = −12→12

### Refinement

**Table:**

Refinement on *F*^2^	Secondary atom site location: difference Fourier map
Least-squares matrix: full	Hydrogen site location: difference Fourier map
*R*[*F*^2^ > 2σ(*F*^2^)] = 0.020	All H-atom parameters refined
*wR*(*F*^2^) = 0.051	*w* = 1/[σ^2^(*F*_o_^2^) + (0.0285*P*)^2^] where *P* = (*F*_o_^2^ + 2*F*_c_^2^)/3
*S* = 1.00	(Δ/σ)_max_ = 0.001
1532 reflections	Δρ_max_ = 0.53 e Å^−^^3^
134 parameters	Δρ_min_ = −1.64 e Å^−^^3^
10 restraints	Absolute structure: Flack (1983), 716 Friedel pairs
Primary atom site location: structure-invariant direct methods	Flack parameter: 0.034 (19)

### Special details

**Table:**

Geometry. All e.s.d.'s (except the e.s.d. in the dihedral angle between two l.s. planes) are estimated using the full covariance matrix. The cell e.s.d.'s are taken into account individually in the estimation of e.s.d.'s in distances, angles and torsion angles; correlations between e.s.d.'s in cell parameters are only used when they are defined by crystal symmetry. An approximate (isotropic) treatment of cell e.s.d.'s is used for estimating e.s.d.'s involving l.s. planes.
Refinement. Refinement of *F*^2^ against ALL reflections. The weighted *R*-factor *wR* and goodness of fit *S* are based on *F*^2^, conventional *R*-factors *R* are based on *F*, with *F* set to zero for negative *F*^2^. The threshold expression of *F*^2^ > σ(*F*^2^) is used only for calculating *R*-factors(gt) *etc*. and is not relevant to the choice of reflections for refinement. *R*-factors based on *F*^2^ are statistically about twice as large as those based on *F*, and *R*- factors based on ALL data will be even larger.

### Fractional atomic coordinates and isotropic or equivalent isotropic displacement parameters (Å^2^)

**Table:**

	*x*	*y*	*z*	*U*_iso_*/*U*_eq_	
Ce1	0.98063 (4)	0.706775 (17)	0.56430 (4)	0.00585 (8)	
Cl1	0.6202 (2)	0.82445 (12)	0.37031 (14)	0.0130 (2)	
B1	0.1685 (9)	0.4466 (5)	0.6439 (6)	0.0073 (10)	
B2	0.8287 (9)	0.4064 (5)	0.6939 (6)	0.0085 (10)	
B3	0.5112 (9)	0.5338 (5)	0.6256 (6)	0.0068 (10)	
B4	0.5809 (9)	0.7447 (5)	0.7169 (6)	0.0062 (10)	
O1	0.9639 (5)	0.4852 (3)	0.6324 (3)	0.0076 (7)	
O2	0.7362 (6)	0.8358 (3)	0.6858 (4)	0.0086 (7)	
O3	0.6294 (6)	0.6361 (3)	0.6426 (4)	0.0093 (7)	
O4	0.3652 (6)	0.7819 (3)	0.6635 (4)	0.0068 (7)	
O5	0.2933 (6)	0.5383 (3)	0.6124 (4)	0.0087 (7)	
O6	0.6027 (6)	0.4259 (3)	0.6207 (4)	0.0086 (7)	
O7	0.6335 (7)	0.7247 (3)	0.8706 (4)	0.0116 (7)	
O8	0.8688 (6)	0.4269 (3)	0.8477 (4)	0.0101 (7)	
H1	0.894 (13)	0.495 (3)	0.876 (7)	0.04 (2)*	
H2	0.731 (13)	0.706 (6)	0.940 (7)	0.04 (2)*	

### Atomic displacement parameters (Å^2^)

**Table:**

	*U*^11^	*U*^22^	*U*^33^	*U*^12^	*U*^13^	*U*^23^
Ce1	0.00577 (12)	0.00558 (12)	0.00601 (12)	0.00003 (13)	0.00121 (8)	0.00002 (14)
Cl1	0.0132 (6)	0.0155 (6)	0.0094 (6)	0.0047 (5)	0.0012 (5)	−0.0001 (5)
B1	0.004 (2)	0.009 (2)	0.009 (3)	−0.0008 (19)	0.001 (2)	−0.002 (2)
B2	0.009 (3)	0.006 (2)	0.009 (3)	0.001 (2)	0.002 (2)	−0.001 (2)
B3	0.011 (3)	0.005 (2)	0.005 (2)	0.001 (2)	0.0029 (19)	0.0021 (19)
B4	0.009 (3)	0.003 (2)	0.006 (2)	0.003 (2)	0.002 (2)	−0.0001 (19)
O1	0.0040 (16)	0.0072 (16)	0.0124 (17)	0.0020 (13)	0.0036 (14)	0.0006 (13)
O2	0.0079 (18)	0.0045 (16)	0.0131 (19)	0.0007 (14)	0.0025 (14)	−0.0005 (14)
O3	0.0083 (18)	0.0063 (15)	0.0145 (18)	0.0011 (14)	0.0055 (14)	−0.0014 (14)
O4	0.0061 (17)	0.0036 (16)	0.0105 (19)	0.0009 (12)	0.0015 (14)	−0.0010 (12)
O5	0.0046 (16)	0.0042 (15)	0.0180 (18)	−0.0009 (13)	0.0041 (14)	0.0023 (14)
O6	0.0027 (15)	0.0077 (14)	0.0138 (17)	−0.0020 (13)	−0.0004 (13)	−0.0019 (13)
O7	0.0091 (19)	0.0155 (17)	0.0097 (19)	0.0059 (15)	0.0017 (15)	0.0023 (15)
O8	0.0161 (18)	0.0084 (17)	0.0048 (17)	−0.0029 (14)	0.0007 (14)	−0.0010 (13)

### Geometric parameters (Å, °)

**Table:**

Ce1—O7^i^	2.480 (4)	B1—O5	1.396 (6)
Ce1—O8^ii^	2.539 (4)	B2—O4^viii^	1.464 (6)
Ce1—O4^iii^	2.579 (4)	B2—O8	1.472 (6)
Ce1—O1	2.589 (3)	B2—O6	1.474 (6)
Ce1—O6^iv^	2.603 (3)	B2—O1	1.483 (7)
Ce1—O2	2.654 (4)	B3—O6	1.359 (6)
Ce1—O3	2.716 (4)	B3—O3	1.370 (6)
Ce1—O5^iii^	2.731 (3)	B3—O5	1.392 (7)
Ce1—Cl1^v^	2.9041 (14)	B4—O4	1.427 (6)
Ce1—Cl1	2.9168 (14)	B4—O7	1.465 (6)
B1—O2^vi^	1.349 (6)	B4—O3	1.496 (6)
B1—O1^vii^	1.378 (6)	B4—O2	1.526 (7)
			
O7^i^—Ce1—O8^ii^	68.55 (12)	O8^ii^—Ce1—Cl1	73.91 (9)
O7^i^—Ce1—O4^iii^	68.81 (13)	O4^iii^—Ce1—Cl1	129.17 (8)
O8^ii^—Ce1—O4^iii^	122.93 (12)	O1—Ce1—Cl1	121.47 (8)
O7^i^—Ce1—O1	123.18 (11)	O6^iv^—Ce1—Cl1	81.76 (8)
O8^ii^—Ce1—O1	67.76 (11)	O2—Ce1—Cl1	64.30 (8)
O4^iii^—Ce1—O1	108.75 (10)	O3—Ce1—Cl1	73.81 (8)
O7^i^—Ce1—O6^iv^	72.87 (12)	O5^iii^—Ce1—Cl1	149.66 (8)
O8^ii^—Ce1—O6^iv^	137.83 (11)	Cl1^v^—Ce1—Cl1	134.62 (5)
O4^iii^—Ce1—O6^iv^	53.00 (10)	Ce1^ix^—Cl1—Ce1	126.35 (5)
O1—Ce1—O6^iv^	152.63 (11)	O2^vi^—B1—O1^vii^	123.2 (5)
O7^i^—Ce1—O2	125.41 (12)	O2^vi^—B1—O5	125.8 (5)
O8^ii^—Ce1—O2	128.40 (12)	O1^vii^—B1—O5	110.9 (4)
O4^iii^—Ce1—O2	106.95 (11)	O4^viii^—B2—O8	111.2 (4)
O1—Ce1—O2	109.92 (11)	O4^viii^—B2—O6	103.8 (4)
O6^iv^—Ce1—O2	64.77 (11)	O8—B2—O6	110.9 (4)
O7^i^—Ce1—O3	147.45 (13)	O4^viii^—B2—O1	110.1 (4)
O8^ii^—Ce1—O3	89.01 (12)	O8—B2—O1	110.8 (4)
O4^iii^—Ce1—O3	142.92 (12)	O6—B2—O1	109.9 (4)
O1—Ce1—O3	63.28 (10)	O6—B3—O3	121.1 (5)
O6^iv^—Ce1—O3	116.78 (11)	O6—B3—O5	118.4 (5)
O2—Ce1—O3	52.04 (11)	O3—B3—O5	120.6 (4)
O7^i^—Ce1—O5^iii^	85.14 (13)	O4—B4—O7	111.1 (4)
O8^ii^—Ce1—O5^iii^	76.69 (11)	O4—B4—O3	112.1 (4)
O4^iii^—Ce1—O5^iii^	63.61 (10)	O7—B4—O3	110.4 (4)
O1—Ce1—O5^iii^	50.80 (10)	O4—B4—O2	111.9 (4)
O6^iv^—Ce1—O5^iii^	116.61 (11)	O7—B4—O2	108.6 (4)
O2—Ce1—O5^iii^	144.19 (12)	O3—B4—O2	102.5 (4)
O3—Ce1—O5^iii^	113.23 (11)	B1^iii^—O1—B2	116.4 (4)
O7^i^—Ce1—Cl1^v^	137.84 (10)	B1^iv^—O2—B4	119.9 (4)
O8^ii^—Ce1—Cl1^v^	136.70 (9)	B3—O3—B4	124.1 (4)
O4^iii^—Ce1—Cl1^v^	69.13 (9)	B4—O4—B2^x^	113.8 (4)
O1—Ce1—Cl1^v^	69.06 (8)	B3—O5—B1	126.3 (4)
O6^iv^—Ce1—Cl1^v^	84.35 (8)	B3—O6—B2	120.5 (4)
O2—Ce1—Cl1^v^	70.67 (8)	B4—O7—H2	143 (8)
O3—Ce1—Cl1^v^	74.57 (8)	Ce1^xi^—O7—H2	80 (8)
O5^iii^—Ce1—Cl1^v^	73.87 (8)	B2—O8—H1	117 (5)
O7^i^—Ce1—Cl1	77.35 (10)	Ce1^xii^—O8—H1	107 (5)

Symmetry codes: (i) *x*+1/2, −*y*+3/2, *z*−1/2; (ii) *x*, −*y*+1, *z*−1/2; (iii) *x*+1, *y*, *z*; (iv) *x*+1/2, *y*+1/2, *z*; (v) *x*+1/2, −*y*+3/2, *z*+1/2; (vi) *x*−1/2, *y*−1/2, *z*; (vii) *x*−1, *y*, *z*; (viii) *x*+1/2, *y*−1/2, *z*; (ix) *x*−1/2, −*y*+3/2, *z*−1/2; (x) *x*−1/2, *y*+1/2, *z*; (xi) *x*−1/2, −*y*+3/2, *z*+1/2; (xii) *x*, −*y*+1, *z*+1/2.

### Hydrogen-bond geometry (Å, °)

**Table:**

*D*—H···*A*	*D*—H	H···*A*	*D*···*A*	*D*—H···*A*
O8—H1···O1^xii^	0.82 (2)	2.43 (7)	2.859 (5)	114 (6)
O8—H1···Cl1^v^	0.82 (2)	2.52 (5)	3.219 (4)	145 (7)
O7—H2···O4^v^	0.83 (2)	2.13 (6)	2.860 (6)	148 (10)
O7—H2···Cl1^v^	0.83 (2)	2.81 (11)	3.220 (4)	112 (9)

Symmetry codes: (xii) *x*, −*y*+1, *z*+1/2; (v) *x*+1/2, −*y*+3/2, *z*+1/2.
